# When happiness strengthens engagement and performance: the role of happiness at work as a resource for experienced employees and newcomers

**DOI:** 10.3389/fpsyg.2025.1560010

**Published:** 2025-07-29

**Authors:** Patrik Fröhlich, Elvira Radaca, Stefan Diestel

**Affiliations:** Schumpeter School of Business and Economics, University of Wuppertal, Wuppertal, Germany

**Keywords:** happiness at work, work engagement, interest-taking, adaptive performance, organizational citizenship behavior (OCB), newcomers, experienced employees

## Abstract

This research investigates the interactive effects of happiness at work and interest taking on work engagement and extra-productive behaviors across experienced employees and newcomers.

**Objective:**

The article investigates how happiness at work can serve as a key resource in enhancing work engagement and extra-productive behaviors such as adaptive performance and organizational citizenship behavior (OCB) in newcomers and experienced employees. Furthermore, it clarifies the interactive effects of the personal resource interest-taking in enhancing the effectiveness of happiness at work, particularly by examining how interest-taking moderates the relationship between happiness at work and work engagement.

**Methods:**

We conducted two longitudinal studies among newcomers (*N* = 126) and experienced employees (*N* = 126) of various industries. For data analysis, we applied multilevel modeling to account for the nested structure of the data. We conducted 2-1-1 moderated mediation analyses to test indirect and conditional effects along the a-path of the proposed model.

**Results:**

Across both studies, our findings indicate indirect effects of happiness at work on extra-productive behavior via work engagement. Interest-taking strengthens the impact of happiness at work on work engagement for newcomers but not for experienced employees. These new insights into the relationship between happiness at work, work engagement, and extra-productive behavior can aid organizations in enhancing the performance and motivation of employees.

**Conclusion:**

Our findings demonstrate that happiness at work indirectly promotes OCB and adaptive performance through increased work engagement for all employees, regardless of their tenure. The moderating role of interest-taking is especially relevant for newcomers, suggesting that organizations can boost positive outcomes by fostering happiness at work in early tenure.

## 1 Introduction

In today's job market, companies face the challenge of attracting and retaining skilled employees in the “War for Talents” era. Here, it is crucial to identify and promote factors that constitute motivating environments to enhance employee motivation and extra-productive behavior, enabling organizations to attract and retain highly talented employees (e.g., Cheese, 2008; Monteiro et al., 2020; Allan et al., 2019; Ehresmann and Badura, 2018). Rehwaldt and Kortsch (2022) suggest that happiness at work (HAW) can be an effective approach to motivating employees in their work. HAW has been linked to extra-productive behaviors such as adaptive performance and organizational citizenship behavior (OCB) (Salas-Vallina et al., 2017; Singh and Banerji, 2022). These behaviors, reflecting proactive adjustment and voluntary contributions beyond formal roles (Griffin et al., 2007; Organ, 1997), are essential for organizational effectiveness (Neuberger, 2006). Drawing on the Job Demands-Resources (JD-R) model (Bakker and Demerouti, 2007, 2017), this study investigates the impact of HAW on adaptive performance and OCB, with work engagement as a mediator and the role of potential moderators. Thereby, organizations' workforces include employees at different stages of their careers, both new and experienced. Newcomers undergo a volatile phase during organizational socialization while they acquire the knowledge, skills, and attitudes required for the new role they adjust to (van Maanen and Schein, 1979) and go from being organizational outsiders to becoming insiders (Bauer et al., 2007). Organizational insiders are characterized by higher levels of knowledge and expertise. They possess a deeper understanding of their job and the organization and are, therefore, called veterans or experienced employees (Bauer and Erdogan, 2011). In line with common practice in socialization literature, for this study, we differentiate between newcomers who have recently been hired, with an organizational tenure of < 1 year, and experienced employees with an organizational tenure of 1 year or longer. We implement a two-study design to examine both target groups and consider employees at different stages of their organizational careers. Hereby, we enlarge the scope of mechanisms of HAW by investigating the proposed effects among experienced employees and newcomers. Based on these two samples, we examine the indirect effects of HAW on adaptive performance and OCB via work engagement in both target groups. Additionally, we seek to uncover interaction effects that further explain the relationship between HAW and work engagement by proposing interest-taking as an amplifying moderator of the positive relationship. Interest-taking is a personal trait that describes the ability to openly reflect on inner and outer circumstances with an unbiased opinion, which creates a state of self-directed awareness of things of inner interest (Ryan and Deci, 2008; Weinstein et al., 2012). Accordingly, it can be assumed that people with a higher level of interest-taking are sensitized to their working environment and, therefore, develop a more precise and more profound feeling for their working environment.

**Figure 1:**
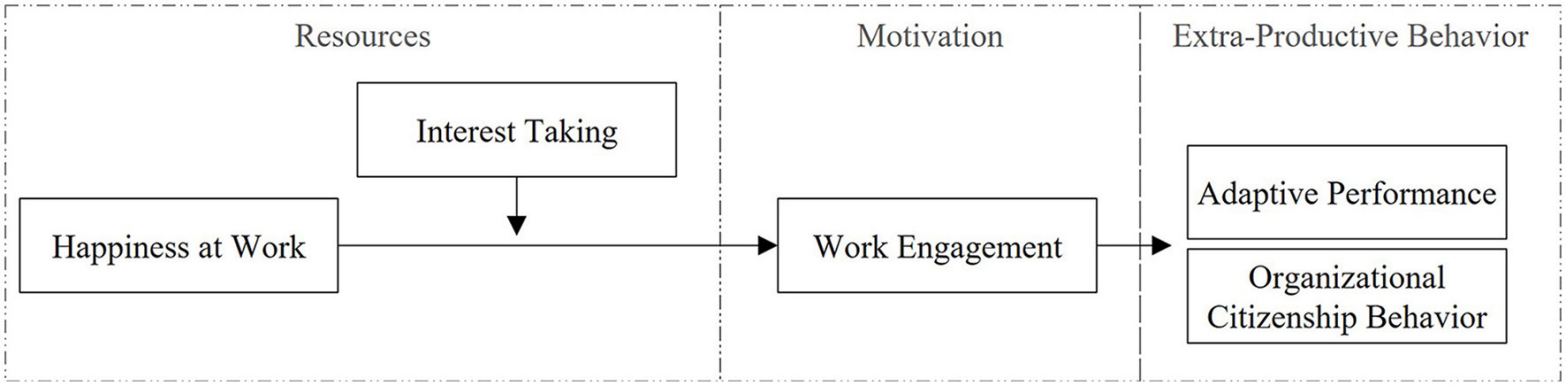
Conceptual research model.

In sum, we propose a model of moderated mediation wherein HAW interacts with interest-taking in predicting work engagement (i.e., we expect interest-taking to enhance the effect), resulting in adaptive performance and OCB (see Figure 1). To investigate our hypotheses among our samples with experienced employees and organizational newcomers, we apply a multilevel analysis of a 2-1-1 moderated mediation model for our studies. We offer several contributions by outlining the role of HAW as a resource in the JD-R model framework and outlining its relevance for newcomers and experienced employees. First, our study uncovers that HAW is an important job-related resource (Bakker and Demerouti, 2007) and indirectly positively affects adaptive performance and OCB. Second, concerning the literature on organizational socialization specifically, our study adds to the knowledge about the role of work engagement for newcomer extra-productive behavior and what resources can help enhance newcomer engagement (Saks and Gruman, 2018, 2012). Third, by including interest-taking as a trait and implementing the person x situation approach (Bakker et al., 2023), we contribute to understanding how personality traits moderate the relationship between the job resource HAW and employee work engagement in different career stage contexts. Concerning practice, human resources management can draw from our insights, which is why we offer practical recommendations on actions and strategies regarding interventions to create motivating work environments with factors relating to HAW.

## 2 Theoretical background

### 2.1 Happiness at work and performance: the mediating role of work engagement

#### 2.1.1 Happiness at work as a resource and its relation to extra-productive behavior

Several studies have shown that work environments and conditions significantly impact psychological wellbeing and extra-productive behavior (e.g., Rossberg et al., 2004). In modern workplaces, factors related to HAW are becoming increasingly relevant for experienced employees and newcomers. HAW can be described as an ideal and positive state that includes evaluations of affective and cognitive components at the workplace (Rehwaldt, 2017). Whereas, various concepts and instruments reflect general happiness and related constructs that refer to broader wellbeing factors (e.g., Butler and Kern, 2016; Su et al., 2014), this conceptualization refers to happiness in the work context (Rehwaldt and Kortsch, 2022). Based on a grounded theory approach by Rehwaldt (2017) and Rehwaldt and Kortsch (2022) propose three central factors that constitute HAW: Meaningfulness, self-actualization, and community. Meaningfulness involves perceiving one's contribution to a meaningful goal and aligning values and goals for organizational coherence. It extends beyond task purposes, encompassing a broader perspective of contributing to a larger purpose and assisting others. Self-actualization entails utilizing personal strengths and abilities to implement ideas at work, driven by individual ideals and beliefs. This leads to increased emotional attachment and commitment, fostering happiness. The third factor, community, is fostered through social interaction and cohesion among members sharing a common goal. It encompasses task-related and professional exchanges and emotional interactions built on trust and familiarity, enhancing the sense of belonging and overall HAW.

Rehwaldt (2017) describes these factors of HAW as a valuable job resource. In general, resources include any means an individual perceives that helps them achieve their goals (Halbesleben et al., 2014). The JD-R model is a theoretical framework to explain the relationship between job demands, resources, and employee wellbeing and performance (Bakker and Demerouti, 2017; Bakker et al., 2023). It distinguishes between two types of resources: Personal resources and job resources. Personal resources are individuals' positive self-evaluations about aspects of themselves associated with personal resilience (Hobfoll et al., 2003) and reflect their beliefs about successfully controlling and influencing their environment (Bakker and Demerouti, 2007). Job resources include different aspects of the job that might lower job demands and associated costs, and/or support employees in achieving work goals, and/or help individuals in their personal development, growth, or learning (Bakker and Demerouti, 2007, 2017). HAW implies that individuals can self-realize and sense the purpose of their work within a trustful and professional community, thus emphasizing aspects of the job that support individuals and help employees grow and succeed. This aligns with the JD-R definition of job resources. Furthermore, HAW is embedded in the framework of eudaimonic wellbeing as it results from “intrinsically motivated, active, and self-determined activities” (Rehwaldt, 2017, p. 83). The eudaimonic concept focuses on factors that contribute to growth and a meaningful work life. In contrast, the hedonic concept focuses on the overall experience of affects, satisfaction, and emotions.^1^ HAW appears variably within investigations using the JD-R model to explain its relationships. Studies in the work context that refer to happiness in the hedonic framework, often using a broader concept of subjective wellbeing, tend to position HAW as an outcome shaped by resources and—to a lesser extent—demands (e.g., Bakker and Oerlemans, 2016; Rodríguez-Muñoz and Sanz-Vergel, 2013; Hafeez et al., 2024; Marsh et al., 2023) or as a mediator between resources, demands and outcomes (e.g., Thompson and Bruk-Lee, 2021). Studies that consider HAW as eudaimonic within the JD-R model framework have begun to position HAW as a resource and investigate its effects on performance (e.g., Salas-Vallina et al., 2020). Thus, in accordance with the eudaimonic framework and the JD-R model, we define HAW as a job resource that comprises intrinsic factors.

We investigate how HAW as a job resource can impact performance via motivation and engagement. Resources are primarily related to a motivational process that can foster organizational outcomes through motivation and engagement, while job demands primarily relate to negative consequences regarding stress and strain through a health impairment process (Bakker and Demerouti, 2024). Therefore, we focus on the motivational process of the JD-R model regarding the relationship of HAW as a resource with work engagement and performance in the remainder of this article.

Previous research demonstrates that employees benefit from higher resources in the form of HAW, as they tend to be more productive and energized, take fewer sick days, intend to stay longer with the organization (Pryce-Jones and Lindsay, 2014) and perform more innovative behaviors (Fröhlich et al., 2025b). Here, Rehwaldt (2017) argues that improving factors that contribute to HAW is not only a goal in itself but also has a significant impact on individual extra-productive behavior and employee engagement. This shows that employees who find meaning in their work, can develop themselves further, and work in an environment where shared goals are pursued are generally more productive, more motivated, and have less absenteeism due to illness (Rossberg et al., 2004; Bashir et al., 2020; Baruch-Feldman et al., 2002). Previous studies consistently demonstrate that HAW is important for employees' wellbeing and extra-productive behavior (e.g., Salas-Vallina et al., 2017; Singh and Banerji, 2022; Fröhlich et al., 2025a). Thus, higher resources in the form of HAW relate to a motivating environment for employees, positively impacting extra-productive behaviors such as adaptive performance and OCB.

OCB refers to employee behaviors that go beyond formal duties of core job tasks and support the social structure of organizations (Fox et al., 2012; Organ, 1997), thus helping organizations as a whole and individuals within the organization (Spector et al., 2010). Those behaviors enhance organizational productivity and include, for example, supporting colleagues or performing beneficial extra-role behaviors. Adaptive performance describes the ability of employees to adapt to new or unforeseen situations successfully and to exhibit appropriate behaviors to deal successfully with these challenges (Jundt et al., 2015). It encompasses employee flexibility and adaptivity in reacting to work-related changes and is an important factor in enhancing individual performance and organizational productivity (Griffin et al., 2007). Both adaptive performance and OCB are crucial for organizations in improving organizational effectiveness, enhancing teamwork and collaboration, and promoting a positive corporate culture (e.g., Podsakoff et al., 2000; Chiaburu et al., 2022).

In summary, HAW is an important resource that enhances employees' extra-productive behaviors, such as adaptive performance and OCB. Higher HAW should help employees better adapt to changes and motivate them to engage in behavior beyond their formal duties, that is, OCB. Nevertheless, only scarce evidence exists on the relationship of HAW with adaptive performance and OCB. In line with the JD-R model and addressing this research gap, we expect HAW as a job resource to positively affect employees' extra-productive behavior in the form of adaptive performance and OCB. We, therefore, propose the following hypotheses:

Hypothesis 1.1: HAW is positively related to adaptive performance among (a) experienced employees (b) and newcomers.
Hypothesis 1.2: HAW is positively related to OCB among (a) experienced employees (b) and newcomers.

#### 2.1.2 The mediating role of work engagement

Focusing on the relationship of resources with individual and organizational outcomes, the motivational path of the JD-R model describes a mediation process via motivation and engagement. It assumes that personal and job resources are positively related to work engagement, which impacts individual behavior and organizational outcomes (Bakker and Demerouti, 2007, 2017). In general, job resources relate to organizational outcomes via work engagement, whereas job demands lead to impairment processes (Bakker and Demerouti, 2024). Although some research suggests that resources can help in dealing with job demands (e.g., Radaca and Diestel, 2025), we primarily focus on the motivational path of the JD-R model. Accordingly, work engagement should mediate the relationship between the job resource of HAW and employee extra-productive behavior. Due to its conceptual role within the JD-R model, most work engagement research deals with either antecedents and consequences of work engagement or its mediating role (e.g., Christian et al., 2011; Lesener et al., 2019; Borst et al., 2020). Work engagement is a positive, fulfilling, motivational state of mind that reflects in vigor (i.e., high level of energy, resilience, and perseverance), dedication (i.e., experiencing a strong involvement, a sense of significance and enthusiasm), and absorption (i.e., being fully absorbed and concentrated in one's work so that time passes quickly (Schaufeli et al., 2002). Employees provided with a work environment that fulfills their expectations have higher levels of engagement (Green et al., 2017). A work environment that reflects factors of HAW will, therefore relate to higher levels of work engagement. Employees who are provided with opportunities to self-actualize and to find meaning in their work will be more engaged and motivated, as they should be better able to sense the significance in what they do, be happily engrossed in their meaningful work and experience higher levels of vitality and perseverance in supportive and trusting community. In a resource-rich work environment characterized by HAW, employees' willingness to dedicate themselves to work will increase (Bakker et al., 2011; Meijman and Mulder, 1998). Therefore, in line with the JD-R model, we expect HAW as a job resource to positively relate to work engagement.

Regarding its relation to extra-productive behavior, several scholars found work engagement essential in predicting adaptive performance (e.g., Costa et al., 2016; Park et al., 2020; Kaya and Karatepe, 2020) and OCB (e.g., Mathumbu and Dodd, 2013; Sulea et al., 2012; Gupta et al., 2017). Meta-analytic evidence demonstrates that work engagement strongly relates to extra-role performance (Borst et al., 2020) and, among various behavioral outcomes, its highest correlation is with OCB (Kanjanakan et al., 2021). Employees who experience increased work engagement report higher levels of vigor, dedication, and absorption at work and will thus be more likely to demonstrate extra-productive behaviors. Regarding adaptive performance, engaged employees are more focused and engrossed in their work (Breevaart et al., 2014), enabling them to detect changes more efficiently and be more ready and dedicated to adapting to those successfully. Therefore, engaged employees should be more likely to demonstrate behaviors that reflect their adaptive performance. Furthermore, regarding OCB, work engagement is positively related to extra-role behaviors (e.g., Eldor and Harpaz, 2016). Engaged employees are more likely to demonstrate OCB as they are dedicated to achieving their work goals while having an increased capability to perform behaviors that go beyond formal work tasks and benefit the organization and individuals within it (Christian et al., 2011). Thus, in line with the JD-R model and former empirical evidence, we expect work engagement to relate positively to both adaptive performance and OCB.

In summary, both the JD-R model and evidence underscore that work engagement is an important mediator between job resources and performance (e.g., Lesener et al., 2019; Neuber et al., 2022; Saks, 2019), strongly suggesting mediation of the relationship between HAW and adaptive performance or OCB. While the vast majority of studies have investigated work engagement among experienced employees, recent research indicates that the mediating role of work engagement in predicting individual extra-productive behavior and performance also applies to newcomers during organizational socialization (Saks and Gruman, 2018). Accordingly, work engagement plays an important mediating role for all employees, both experienced and new to the organization.

In conclusion, the JD-R model and recent socialization literature suggest that HAW will positively influence work engagement for both experienced employees and newcomers. This assumption is also consistent with the findings of Lesener et al. (2020), as HAW is a resource that is closely connected to the individual and thus has a presumably strong effect on work engagement. Moreover, theory and empirical research have consistently linked work engagement to extra-productive behavior in both populations and demonstrated the mediating role of work engagement between job resources and extra-productive behavior, particularly concerning adaptive performance and OCB. Thus, it is reasonable to assume that work engagement mediates the relationship between HAW and extra-productive behavior in the form of adaptive performance and OCB. Therefore, we postulate the following hypotheses:

Hypothesis 2.1: HAW is positively indirectly related to adaptive performance via work engagement among (a) experienced employees (b) and newcomers.
Hypothesis 2.2: HAW is positively indirectly related to OCB via work engagement among (a) experienced employees and (b) newcomers.

### 2.2 The moderating role of interest-taking

We propose that employees with higher levels of interest-taking, reflecting in attentional self-directed regulation at work and openness to internal and external circumstances, will be better able to benefit from HAW in demonstrating increased work engagement. Drawing from the person x situation approach of the JD-R model (Bakker et al., 2023), we seek to improve understanding of the interaction between an individual's personality and work situation. The approach assumes that an individual's behavior results from their unique personality traits and the specific situational factors they encounter. Considering the effects of personality traits helps improve our understanding of the relationship between HAW and work engagement.

Interest-taking, a central facet of trait autonomy, is the conscious ability to think about and reflect on internal and external circumstances, involving both cognitive and motivational processes that encompass awareness and ongoing insight into oneself and one's experiences, promoting a high degree of self-oriented regulation (Weinstein et al., 2012; Ryan and Deci, 2008). Specifically, this means that employees take an active interest in a given circumstance or thing, building a personal connection to it. This helps them, e.g., stay intrinsically motivated in a task or activity. In addition, in interest-taking, individuals actively reflect on phenomena or conditions as well as circumstances in a curious rather than defensive manner. That is, individuals with high levels of interest-taking are better able to be open to, reflect on, and match internal and external events with their inner selves (Weinstein et al., 2012). The main element of interest-taking is the awareness of one's own experiences and self in these moments (Weinstein et al., 2012, p. 398), which reflects a higher level of self-directed attention. Thus, we assume that interest-taking is crucial in enabling employees to optimally process the conditions and circumstances they face at work, such as factors that determine HAW, and assess the extent to which these align with their self. This leads to a higher degree of self-direction and the ability to leverage these factors and conditions at work, ultimately enhancing work engagement. Complementing this argumentation, we can transfer the expected interaction to the resource-reciprocity proposition of the JD-R model (Bakker et al., 2023). The JD-R model expects that resources reciprocate so that individuals with higher levels of personal resources can access higher levels of job resources and vice versa (Bakker et al., 2023, p. 33), leading to a joint positive impact on work engagement. Interest-taking can be characterized as a personal resource, as it represents a positive self-evaluation related to the ability to control and impact the work environment. In contrast, HAW is referred to as a job resource. That means a higher level of the personal resource of interest-taking should relate to better accessibility of the job resource of HAW, consequently enhancing work engagement.

In summary, employees (newcomers and experienced employees) with higher levels of interest-taking, representing a personal resource, are better able to perceive and profit from a working environment that aligns with their values, feelings, and interests. Therefore, the influence of HAW, which represents a job resource, on work engagement will be enhanced, leading to the following hypothesis:

Hypothesis 3: Interest-taking moderates the positive relationship between HAW and work engagement among (a) experienced employees and (b) newcomers; the relationship will be stronger (weaker) for individuals with higher (lower) interest-taking.

In line with the JD-R model, we propose that higher job resources related to HAW will positively impact work engagement, enhancing adaptive performance and OCB. Thus, we expect work engagement to mediate the respective positive relationship of HAW with adaptive performance and OCB among newcomers and experienced employees. In addition, we expect employees with higher levels of interest-taking, represented by a higher degree of self-directed attention, to be better able to process beneficial conditions and circumstances at work and thus leverage the factors related to HAW, further enhancing their work engagement. Overall, we expect that both indirect effects of HAW on adaptive performance and OCB via work engagement will be stronger (weaker) for individuals with higher (lower) levels of interest-taking. This leads to the following hypotheses:

Hypothesis 4.1: Interest-taking moderates the indirect effect of HAW on adaptive performance via work engagement among (a) experienced employees and (b) newcomers.
Hypothesis 4.2: Interest-taking moderates the indirect effect of HAW on OCB via work engagement among newcomers among (a) experienced employees and (b) newcomers.

## 3 Overview of the studies

As part of this research, we make empirical efforts to replicate the hypothesized results in different samples and different situational contexts. As already evident in our hypotheses, we do not expect different results. Since our two target groups are in different phases and situational contexts, it was essential to adapt the study design to the respective target group accordingly, even if the phenomena studied are assumed to be equally effective. As a consequence, we conducted two studies to test our hypotheses. The first study includes experienced employees from various organizations who participated in a diary study. The second study uses a monthly assessment to focus on organizational newcomers. By expanding the examination of our research model into the domain of organizational socialization among newcomers, we seek to gather insights into commonalities and differences in the interactive effects of HAW and interest-taking on work engagement and performance. By doing so, we also gain insights into how HAW might be important for newcomers and how this relates to enhancing newcomers' work engagement during organizational socialization. Furthermore, we improve the generalizability by replicating our findings among varying samples. For both studies, we use multilevel analysis to test a model of 2-1-1 moderated mediation (Preacher et al., 2010, 2011), with work engagement mediating the effect of HAW on OCB and adaptive performance, and interest taking moderating the effect of HAW on work engagement. This methodological approach allows us to separate within- from between-person effects in investigating the relationships.

## 4 Study 1: experienced employees

### 4.1 Materials and methods

#### 4.1.1 Research design and participants

For this diary study, we recruited experienced employees from diverse occupational backgrounds. Recruitment was performed via convenience sampling, using direct contact and contacts with different companies. Participation in the study was voluntary and without monetary compensation. However, participants received individual feedback on their data upon request. All participants were fully informed about data protection, the purpose of the study, and all procedures before participating.

We conducted this diary study using an online survey. The pre-questionnaire consisted of stable constructs, such as sociodemographic information and person-related variables (e.g., HAW and interest-taking). Over 10 working days (Monday to Friday), participants received three emails a day (morning, noon, and evening) with links to the respective daily questionnaires. The study was suspended on weekends and holidays and resumed on the next regular work day. The timing of the daily questionnaires was based on the participants' self-reported working hours. The first email was sent 2 before the start of work, the second 4 h into the workday, and the last email was sent 1 h after work ended. Participants had 2 h to complete each daily questionnaire. They received a reminder email if they did not complete it within 1 h.

Of the initial 138 participants recruited, 12 were excluded due to incomplete daily questionnaires for at least one reported day. The final sample consisted of 126 employees with fully completed daily questionnaires on an average of 6.37 out of a maximum of 10 survey days, resulting in a total of 803 daily measurement points. All data collected in the daily diary study was self-reported.

65.90% were female, and the average age was 34.20 years (SD = 13.50). The work experience was 13.97 years (SD = 14.32) on average, and the average organizational tenure was 6.45 years (range = 1–43; SD = 9.06). 15.90% of the participants held supervisory positions, and 59.50% were full-time employees (i.e., working 40 h per week). The majority of the participants were from the financial and insurance sector (18.30%), healthcare (10.30%), science (9.50%), IT and communication (8.70%), production and processing industry (7.90%), and miscellaneous industries (21.40%). Consequently, the sample was heterogeneous and comprised individuals with diverse professional and individual backgrounds.

#### 4.1.2 Measures

We assessed HAW and interest-taking in the pre-questionnaire. All other constructs were assessed daily as repeated measures: work engagement as a state at noon, adaptive performance, and OCB in the evening to reflect on the whole working day. See Table 1 for an overview of all measures.

**Table 1:** Measures of focal variables.

**Variable**	**Source**	**Item count**	**Response scale**	**Sample items**
Happiness at work	Rehwaldt and Kortsch, 2022	12	1 (“disagree”) to 5 (“totally agree”)	“I can implement my ideas and wishes”^ab^ “I feel that my work is meaningful.”^ab^ “In our company, we treat each other with respect.”^ab^
Interest-taking	Weinstein et al., 2012	3	1 (“not at all true”) to 5 (“completely true”)	“I often reflect on why I react the way I do.”^ab^
Work engagement	Schaufeli et al., 2006	9	1 (“never”) to 7 (“always”)	“At my work, I feel^a^/felt^b^ bursting with energy.” “My job inspires^a^/inspired^b^ me.” “I am^a^/was^b^ immersed in my work.”
Adaptive performance	Griffin et al., 2007	3	1 (“very little”) to 5^a^/to 7^b^ (“a great deal”)	“I adapted well to changes in my core tasks.”^ab^
OCB	Staufenbiel and Hartz, 2000^a^	7^a^	1 (“does not apply at all”) to 7 (“fully applies”)^a^	“Today, I actively sought to prevent difficulties with colleagues.”^a^
	Spector et al., 2010^b^	10^b^	1 (“never”) to 5 (“every day”)^b^	“Helped a co-worker who had too much to do”^b^ “Offered suggestions to improve how work is done.”^b^

OCB, Organizational Citizenship Behavior. We used the same scales for both studies, except for OCB. For study 1, work engagement was worded as a state, while adaptive performance and OCB reflected the whole working day and were worded accordingly. To account for the retrospective assessment of all the repeated measures in Study 2 (work engagement, adaptive performance and OCB), items were reworded and the instruction was adapted accordingly. ^a^Study 1. ^b^Study 2.

#### 4.1.3 Analytical procedure

All analyses were performed with Mplus 8.7 (Muthén and Muthén, 2017). We applied multilevel path analysis to test our 2-1-1 model of moderated mediation (Preacher et al., 2010, 2011). Following recent recommendations on 2-1-1 multilevel mediation (Fang et al., 2019), we used the Bayesian estimation method that has repeatedly demonstrated better accuracy and efficiency compared to frequentist approaches (e.g., maximum likelihood) for multilevel models that include moderated mediation (Asparouhov and Muthén, 2021b). To estimate the moderated mediation model, we specified a level-2 interaction between the moderator (i.e., interest-taking) and the independent variable (i.e., HAW). For an unbiased estimation, we centered both level-2 variables and their interaction around the grand mean (Enders and Tofighi, 2007). Bayesian estimation is based on the Markov Chain Monte Carlo algorithm, where multiple iterations are used for calculating posterior parameter values (Zyphur and Oswald, 2015). We rely on non-informative priors to allow unbiased inferences (Wang and Preacher, 2015), as our hypotheses include novel relationships. Bayesian estimation does not deliver fixed values with significance values for parameter estimates but instead makes use of the distribution of information for the parameters. Therefore, a credibility interval (CrI), based on the posterior distributions, is provided for each parameter estimate. In a 95% CrI, the effect has a 95% probability of falling within the given range. Thus, similar to the logic of frequentist confidence intervals, including zero in a 95% CrI would indicate that the respective parameter might not differ from zero. For convergence and fit of our respective models in the two studies, we evaluated the potential scale reduction value, trace plots for the distribution, model parameter autocorrelations, and Posterior Predictive *p*-values (Asparouhov and Muthén, 2021a).

### 4.2 Results of study 1

#### 4.2.1 Descriptive statistics, construct validity, and model fit

Table 2 shows means, correlations, intraclass correlations (ICCs), and reliabilities.^2^ We first examined within-person (Level 1) and between-person (Level 2) variances among our outcome variables and evaluated the model fit before testing the hypotheses. A substantial amount of between-person variance was given (see ICC values in Table 2). Thus, the results of variance decomposition strongly support the application of multilevel modeling.

**Table 2:** Descriptive statistics for study 1 (experienced employees).

**Variable**	***M***	**SD**	**ICC**	**Correlations**							
				**1**.	**2**.	**3**.	**4**.	**5**.	**6**.	**7**.	**8**.
1. Work engagement^a^	4.78	1.25	0.69	(0.95)	**0.25**	**0.12**					
2. Adaptive performance^b^	3.20	1.02	0.37	0.13	(0.79)	**0.17**					
3. OCB^a^	3.34	1.30	0.61	0.18	**0.63**	(0.83)					
4. Happiness at work^b^	3.63	0.60		**0.50**	0.16	0.19	(0.82)				
5. Interest-taking^b^	3.42	0.79		−0.04	0.09	0.06	0.17	(0.81)			
6. Age^c^	34.20	13.50		0.09	−0.06	**−0.23**	0.15	−0.15	-		
7. Gender^d^	1.33	0.47		0.12	0.10	0.11	0.04	−0.14	0.72	-	
8. Work experience^c^	13.97	14.32		0.09	−0.04	**-0.18**	0.14	−0.15	**0.96**	0.41	-
9. Organizational tenure^c^	6.45	9.06		−0.10	0.07	−0.11	−0.05	**−0.27**	**0.64**	0.13	**0.69**

OCB, Organizational Citizenship Behavior; M, Grand means for person-level means; SD, Standard deviation of grand means for person-level means; ICC, Intraclass Correlations of within-variables. Values for McDonald's Omega are depicted in parentheses on the diagonal. Below the diagonal are between-level correlations (N = 126), and above the diagonal are within-level correlations (**N** = 803). Numbers in bold = 95% Credibility Interval does not include zero. ^a^7-point scale. ^b^5-point scale. ^c^In years. ^d^1 = female, 2 = male.

We performed an MCFA to examine the construct validity of the self-report measures. A 5-factor model with separate HAW, interest-taking, work engagement, adaptive performance, and OCB was tested against three alternative models. As Table 3 shows, the 5-factor model had a good fit and fitted our data best compared to the alternatives. A supplemental exploratory factor analysis with Harman's single-factor test (Podsakoff et al., 2003) also indicated that there was no single factor at the within or between level that would account for substantial shared variance, suggesting the absence of common method variance.

**Table 3:** Confirmatory factor analyses results for study 1 (experienced employees).

**Model**	**χ^2^ (df)**	***Δχ* ^2^**	**CFI**	**TLI**	**RMSEA**	**SRMR_w_**	**SRMR_b_**
1 Within: work engagement; adaptive performance; OCB Between: happiness at work; interest-taking	723.37 (260)	–	0.93	0.91	0.05	0.06	0.08
2 Within: work engagement and adaptive performance as one factor; OCB Between: happiness at work; interest-taking	1466.33 (265)	742.96^***^	0.81	0.78	0.08	0.02	0.08
3 Within: work engagement; adaptive performance and OCB as one factor Between: happiness at work; interest-taking	1114.06 (262)	390.68^***^	0.86	0.84	0.06	0.07	0.08
4 Within: work engagement and adaptive performance and OCB as one factor Between: happiness at work and interest-taking as one factor	296.39 (271)	2240.01^***^	0.57	0.51	0.11	0.16	0.16

*N*_*between*_= 126, *N*_*within*_= 803. OCB, Organizational Citizenship Behavior; CFI, Comparative Fit Index; TLI, Tucker-Lewis Index; RMSEA, Root Mean Square Error of Approximation. SRMR_*w*_/SRMR_*b*_, Standardized Root Mean Residual for within/between. ^***^p < 0.001.

#### 4.2.2 Test of hypotheses

Before hypothesis testing, the trace plot inspection and a stable potential scale reduction value of < 1.05 after ~200 iterations indicated a very good model convergence. Results further indicated a good fit for the mediation model [95%-CI = [−18.98; 29.75]; Posterior Predictive *P*-Value = 0.33].

For Hypothesis 1.1a and Hypothesis 1.2a, we tested the direct positive effect of HAW on adaptive performance and OCB, respectively. Multilevel estimates do not indicate direct effects of HAW on adaptive performance [*B* = 0.12, 95%-CrI = [−0.13; 0.38]] and OCB [*B* = 0.21, 95%-CrI = [0.16; 0.60]]. Therefore, Hypothesis 1.1a and Hypothesis 1.2a have to be rejected, and HAW does not directly relate to adaptive performance and OCB among experienced employees.

Hypothesis 2.1a proposed the indirect positive effect of HAW on adaptive performance via work engagement. Hypothesis 2.2a proposed an indirect effect of HAW and OCB via work engagement. Consistent with Hypothesis 2.1a and Hypothesis 2.2a, multilevel estimates revealed that between-person HAW positively related to work engagement [*B* = 0.90, 95%-CrI = [0.60; 1.21]]. At the within-person level, work engagement was positively related to both adaptive performance [*B* = 0.30, 95%-CrI = [0.21; 0.38]] and OCB [*B* = 0.14, 95%-CrI = [0.05; 0.23]], supporting Hypothesis 2.1a and Hypothesis 2.2a respectively. Consequently, the two hypothesized indirect effects of HAW via work engagement on adaptive performance [*B* = 0.26, 95%-CrI = [0.16; 0.39]] and OCB [*B* = 0.13, 95%-CrI = [0.05; 0.23]] were evident among experienced employees (see Table 4). Both Hypothesis 2.1a and Hypothesis 2.2a are therefore supported. Thus, work engagement fully mediates the positive relationships between HAW and adaptive performance (Hypothesis 2.1a) and OCB (Hypothesis 2.2a) among experienced employees.

**Table 4:** Multilevel estimates for study 1 (experienced employees).

	**Model 1 (mediation)**				**Model 2 (moderated mediation)**			
**Parameter**	***B***	**PSD**	**95% CrI LL**	**95% CrI UL**	***B***	**PSD**	**95% CrI LL**	**95% CrI UL**
**Within-level**								
Direct effects								
WE → AP	**0.29**	0.04	0.21	0.38	**0.30**	0.04	0.21	0.38
WE → OCB	**0.14**	0.05	0.05	0.23	**0.14**	0.05	0.05	0.23
*R*^2^ AP	**0.06**	0.02	0.03	0.11	**0.07**	0.02	0.03	0.10
*R*^2^ OCB	**0.01**	0.01	0.00	0.04	**0.01**	0.01	0.00	0.04
**Between-level**								
Direct effects								
WE → AP	0.04	0.07	−0.10	0.18	0.04	0.07	−0.11	0.18
WE → OCB	0.11	0.11	−0.11	0.33	0.11	0.11	−0.12	0.32
*R*^2^ WE	**0.23**	0.07	0.11	0.37	**0.26**	0.07	0.13	0.40
*R*^2^ AP	**0.04**	0.04	0.00	0.14	**0.04**	0.04	0.00	0.14
*R*^2^ OCB	**0.05**	0.04	0.00	0.15	**0.05**	0.04	0.00	0.16
**Cross-level**								
Direct effects								
HAW → WE	**0.87**	0.15	0.57	1.17	**0.90**	0.16	0.60	1.21
HAW → AP	0.13	0.13	−0.13	0.37	0.12	0.13	−0.13	0.38
HAW → OCB	0.22	0.19	−0.17	0.60	0.21	0.19	−0.16	0.60
IT → WE					−0.19	0.12	−0.41	0.05
**Indirect effects**								
HAW → WE → AP	**0.25**	0.06	0.15	0.38	**0.26**	0.06	0.16	0.39
HAW → WE → OCB	**0.12**	0.05	0.04	0.23	**0.13**	0.05	0.05	0.23
**Interaction**								
HAW × IT → WE					−0.14	0.17	−0.47	0.19

*N*_*between*_= 126, *N*_*within*_= 803. PSD, Posterior Standard Deviation; HAW, Happiness at Work; WE, Work Engagement; IT, Interest-Taking; AP, Adaptive Performance; OCB, Organizational Citizenship Behavior. 95% CrI LL (UL), Lower (Upper) Level of 95% Credibility Interval. Bold values indicate parameters' 95% Credibility Interval does not include zero.

In Hypothesis 3a, we predicted moderating effects (amplifying effects) of interest-taking on the positive relationship between HAW and work engagement among experienced employees. However, multilevel estimates do not indicate an interaction effect of HAW × interest-taking on work engagement [*B* = −0.14, 95%-CrI = [−0.47; 0.19]]. Therefore, Hypothesis 3a did not receive support from the first sample's data. Interest-taking does not moderate the positive relationship between HAW and work engagement among experienced employees. Consequently, the proposed moderator's conditional indirect effects at higher or lower levels could not be interpreted. Thus, Hypothesis 4.1a and Hypothesis 4.2a are not supported.

#### 4.2.3 Supplementary analysis

Although the current study focused on the moderating role of interest-taking, we conducted supplementary analyses to examine the other subscales of the index of autonomous functioning. Multilevel estimation revealed non-existent interactions of the sub-facets of susceptibility to control [*B* = 0.01; 95%-CrI = [−0.16; 0.17]] and authorship [*B* = −0.03; 95%-CrI = [−0.56; 0.50]] with HAW. Therefore, none of the subscales of the index of autonomous functioning moderated the positive effect of HAW on work engagement among experienced employees.

### 4.3 Discussion of study 1

In our first study, we sought to investigate the mediating role of work engagement in a sample of experienced employees, and our findings confirmed our hypothesis. Our results indicate that HAW had a positive effect on work engagement, which in turn positively influenced extra-role performance in the form of adaptive performance and OCB. The path of motivation triggered by HAW highlights the importance of this construct as a key resource for experienced employees. Although we initially hypothesized that interest-taking moderates the positive effect of HAW on work engagement, our first study does not provide evidence for this interaction. One possible explanation for this lack of moderation is that experienced employees often possess a deep understanding of work processes and company culture and are frequently capable of adapting their work to their interests and skills. Furthermore, their experience often allows them to quickly acclimate to new tasks, which might limit the relevance of interest-taking in moderating the relationship between HAW and work engagement. However, our study's findings provide valuable insights into the mediating role of work engagement between HAW and extra-role performance among experienced employees.

## 5 Study 2: newcomers

### 5.1 Materials and methods

#### 5.1.1 Research design and participants

We recruited newcomers from various organizations in Germany using the convenience sampling method through professional networks or direct contact. Participants self-registered for the online study via a double opt-in email procedure. All participants were fully informed about the study's details and assured of data confidentiality and security. Participation was voluntary, and participants received no monetary compensation. However, as in Study 1, participants were given the option of receiving individual feedback. Of the 246 people who initially participated in the survey, 120 were excluded because they had only completed one survey or because data were either illogical or incomplete. The final sample included 126 newcomers. The average number of completed surveys was 3.17, resulting in a total of 399 observations. The average age was 27.84 years (SD = 6.73 years). 63.50% of the sample were female. Work experience was 4.26 years on average (SD = 6.48), and the average number of previous job changes was 2.15 (SD = 2.17), reflecting the participants' experience with socialization processes. 10.30% of the newcomers were in leadership positions, and 31.70% worked part-time. Participants came from various industries: The health and social care sector (18.90%), service industry (17.10%), wholesale and retail (16.20%), education and upbringing (12.60%), or information and communications (8.10%). Thus, we investigated a heterogeneous sample of newcomers with a wide range of professional and personal backgrounds. Participants filled out the first questionnaire 2–4 weeks after organizational entry, covering the time since they started their job. The three consecutive questionnaires were then sent at 4-week intervals, covering the initial 4 months of the new employment.

#### 5.1.2 Measures

We used the same scales as in Study 1, except for OCB (see Table 1). The first questionnaire included demographic information, such as age or gender, and measures of HAW and interest-taking as a trait. The first and the subsequent three questionnaires assessed work engagement, adaptive performance, and OCB.

#### 5.1.3 Analytical procedure

The procedures of Study 1 were adopted accordingly.

### 5.2 Results of study 2

#### 5.2.1 Descriptive statistics, construct validity, and model fit

Table 5 displays descriptive statistics and reliabilities of variables. Similar to Study 1,^3^ examination for within-person (Level 1) and between-person (Level 2) variances among the outcome variables revealed substantial amounts of variance on Level 2 (see ICC values in Table 5). In line with Study 1, the results of variance decomposition also support the application of multilevel modeling.

**Table 5:** Descriptive statistics for study 2 (newcomers).

**Variable**	***M***	***SD***	**ICC**	**Correlations**							
				**1**.	**2**.	**3**.	**4**.	**5**.	**6**.	**7**.	**8**.
1. Work engagement^a^	4.64	1.21	0.71	(0.95)	**0.34**	**0.18**					
2. Adaptive performance^a^	5.43	0.95	0.43	**0.55**	(0.75)	**0.14**					
3. OCB^b^	2.53	0.70	0.63	**0.34**	**0.26**	(0.82)					
4. Happiness at work^b^	3.68	0.70		**0.67**	**0.46**	0.26	(0.89)				
5. Interest-taking^b^	3.49	0.72		**0.22**	0.18	−0.09	0.22	(0.77)			
6. Age^c^	27.84	6.73		0.16	0.11	0.17	0.07	−0.05	–		
7. Gender^d^	1.37	0.48		−0.08	−0.08	0.12	0.02	−0.06	0.02	–	
8. Work experience^c^	4.26	6.48		0.09	0.09	0.13	−0.03	−0.05	**0.90**	0.02	–
9. Job change experience^e^	2.15	2.17		−0.02	−0.09	0.16	−0.09	−0.09	**0.48**	0.08	**0.58**

OCB, Organizational Citizenship Behavior; M, Grand means for person-level means; SD, Standard deviation of grand means for person-level means; ICC, Intraclass Correlations of within-variables. Values for McDonald's Omega are depicted in parentheses on the diagonal. Below the diagonal are between-level correlations (N = 126), and above the diagonal are within-level correlations (N = 399). Numbers in bold = 95% Credibility Interval does not include zero. ^a^7-point scale. ^b^5-point scale. ^c^In years. ^d^1 = female, 2 = male. ^e^Total number of previous job changes.

As in Study 1, a 5-factor model with separate HAW, interest-taking, work engagement, adaptive performance, and OCB was tested against three alternative models. As can be seen in Table 6, the 5-factor model had a good fit and better fitted our data compared to the alternative models. We could replicate the factor structure from Study 1, and the discriminability of our measures was given. Supplemental exploratory factor analysis again indicated no substantial shared variance at a single factor at the within or between level, again suggesting the absence of common method variance.

**Table 6:** Confirmatory factor analyses results for study 2 (newcomers).

**Model**	**χ^2^ (df)**	***Δχ* ^2^**	**CFI**	**TLI**	**RMSEA**	**SRMR_w_**	**SRMR_b_**
1 Within: work engagement; adaptive performance; OCB Between: happiness at work; interest-taking	733.20 (320)	–	0.91	0.90	0.06	0.06	0.07
2 Within: work engagement and adaptive performance as one factor; OCB Between: happiness at work; interest-taking	920.80 (322)	187.60^***^	0.87	0.85	0.07	0.07	0.07
3 Within: work engagement; adaptive performance and OCB as one factor Between: happiness at work; interest-taking	1028.87 (322)	295.67^***^	0.85	0.83	0.07	0.11	0.07
4 Within: work engagement and adaptive performance and OCB as one factor Between: happiness at work and interest-taking as one factor	1781.58 (328)	1048.38^***^	0.69	0.65	0.11	0.13	0.12

*N*_*between*_= 126, *N*_*within*_= 399. OCB, Organizational Citizenship Behavior; CFI, Comparative Fit Index; TLI, Tucker-Lewis Index; RMSEA, Root Mean Square Error of Approximation; SRMR_*w*_/SRMR_*b*_, Standardized Root Mean Residual for within/between. ^***^p < 0.001.

#### 5.2.2 Test of hypotheses

Trace plot inspection and the potential scale reduction value falling below 1.05 after ~500 iterations indicated good model convergence. Like in Study 1, results revealed a good model fit [95%-CI = [−19.60; 26.47]; Posterior Predictive *P*-Value = 0.41].

Hypothesis 1.1b and Hypothesis 1.2b postulated the respective direct positive effects of HAW on adaptive performance and OCB among newcomers. Multilevel estimates do not confirm the direct effects of HAW on adaptive performance [*B* = 0.14, 95%-CrI = [−0.09; 0.38]] or OCB [*B* = 0.04, 95%-CrI = [−0.17; 0.25]]. Among newcomers, HAW is not directly related to adaptive performance and OCB.

Hypothesis 2.1b and Hypothesis 2.2b proposed that work engagement mediates the positive effect of HAW on newcomer adaptive performance (Hypothesis 2.1b) and newcomer OCB (Hypothesis 2.2b), respectively. The results show that between-person HAW was related to newcomer within-level work engagement [*B* = 1.01, 95%-CrI = [0.79; 1.23]]. On the within-person level, newcomer work engagement was related to adaptive performance [*B* = 0.37, 95%-CrI = [0.25; 0.50]] and OCB [*B* = 0.12, 95%-CrI = [0.04; 0.19]]. Therefore, the results support the indirect effect of HAW on adaptive performance via work engagement [*B* = 0.37, 95%-CrI = [0.24; 0.54]] and the indirect effect of HAW on OCB via work engagement [*B* = 0.12, 95%-CrI = [0.04; 0.20]] (see Table 7). Thus, Hypothesis 2.1b and Hypothesis 2.2b were supported. Consistent Study 1 on experienced employees, work engagement also fully mediates the positive relationship between HAW and adaptive performance (Hypothesis 2.1b) and the positive relationship between HAW and OCB (Hypothesis 2.2b) among newcomers.

**Table 7:** Multilevel estimates for study 2 (newcomers).

**Parameter**	**Model 1 (mediation)**				**Model 2 (moderated mediation)**			
	***B***	**PSD**	**95% CrI LL**	**95% CrI UL**	***B***	**PSD**	**95% CrI LL**	**95% CrI UL**
**Within-level**								
Direct effects								
WE → AP	**0.37**	0.06	0.25	0.50	**0.37**	0.06	0.25	0.50
WE → OCB	**0.12**	0.04	0.04	0.20	**0.12**	0.04	0.04	0.19
R^2^ AP	**0.11**	0.04	0.05	0.20	**0.11**	0.04	0.05	0.19
R^2^ OCB	**0.03**	0.02	0.00	0.09	**0.03**	0.02	0.00	0.08
**Between-level**								
Direct effects								
WE → AP	**0.27**	0.09	0.10	0.44	**0.28**	0.09	0.11	0.44
WE → OCB	**0.17**	0.08	0.01	0.32	**0.17**	0.08	0.02	0.33
R^2^ WE	**0.45**	0.07	0.30	0.59	**0.54**	0.07	0.39	0.66
R^2^ AP	**0.33**	0.09	0.16	0.51	**0.35**	0.09	0.17	0.53
R^2^ OCB	**0.13**	0.06	0.03	0.27	**0.14**	0.07	0.03	0.29
**Cross-level**								
Direct effects								
HAW → WE	**0.98**	0.11	0.77	1.20	**1.01**	0.11	0.79	1.23
HAW → AP	0.15	0.12	−0.09	0.39	0.14	0.12	−0.09	0.38
HAW → OCB	0.05	0.11	−0.16	0.26	0.04	0.11	−0.17	0.25
IT → WE					0.10	0.11	−0.10	0.31
**Indirect effects**								
HAW → WE → AP	**0.36**	0.08	0.23	0.53	**0.37**	0.08	0.24	0.54
HAW → WE → OCB	**0.12**	0.04	0.04	0.20	**0.12**	0.04	0.04	0.20
**Interaction**								
HAW × IT → WE					**0.46**	0.14	0.19	0.72
**Conditional a-path**								
HAW × IT (−1 SD) → WE					**0.67**	0.14	0.40	0.95
HAW × IT (+1 SD) → WE					**1.34**	0.16	1.03	1.65
**Conditional indirect effects**								
HAW × IT (−1 SD) → WE → AP					**0.25**	0.07	0.13	0.40
HAW × IT (+1 SD) → WE → AP					**0.50**	0.10	0.31	0.71
HAW × IT (−1 SD) → WE → OCB					**0.08**	0.03	0.02	0.15
HAW × IT (+1 SD) → WE → OCB					**0.15**	0.06	0.05	0.27

*N*_*between*_= 126, *N*_*within*_= 399; PSD, Posterior Standard Deviation; HAW, Happiness at Work; WE, Work Engagement; IT, Interest-Taking; AP, Adaptive Performance; OCB, Organizational Citizenship Behavior; 95% CrI LL (UL), Lower (Upper) Level of 95% Credibility Interval. Bold values indicate parameters' 95% Credibility Interval does not include zero.

**Figure 2:**
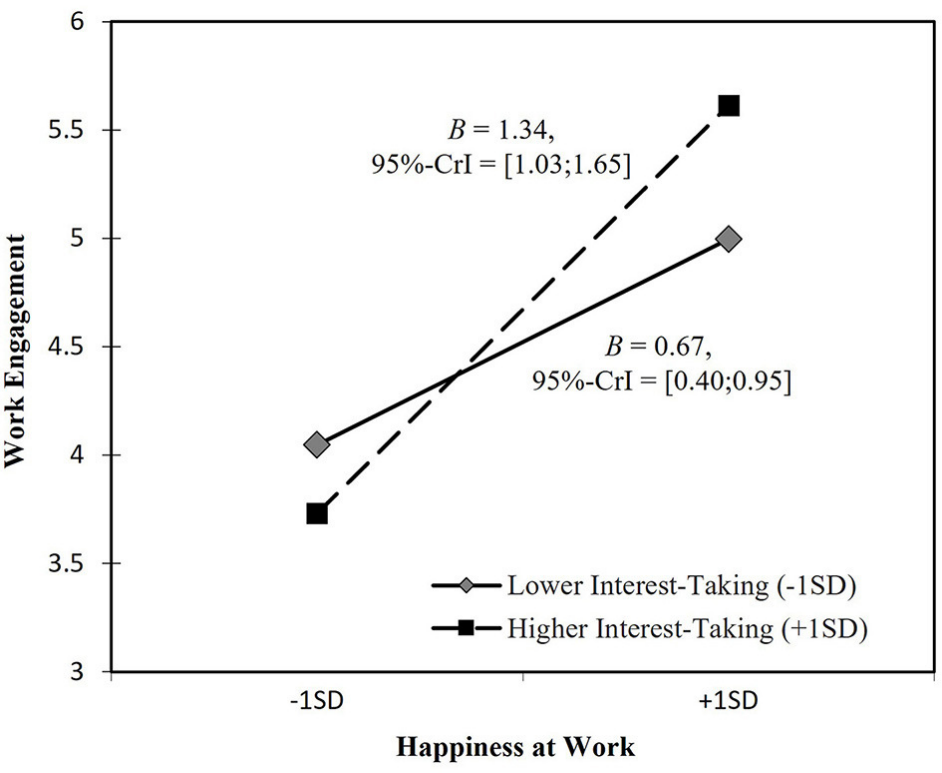
Interaction effects of happiness at work and interest-taking.

Hypothesis 3b addressed the moderating role of interest-taking. It proposes that the positive relationship between HAW and work engagement is stronger for newcomers with higher (vs. lower) interest-taking. In support of this proposition, results indicate that interest-taking moderates the positive relationship between HAW and work engagement [*B* = 0.46, 95%-CrI = [0.19; 0.72]]. We performed a simple slope analysis for values of the moderator at one standard deviation above (+1SD) and below (−1SD) the mean as recommended by Preacher et al. (2006) and depicted the interaction in Figure 2. Interaction patterns show that for newcomers with higher levels of interest-taking, the positive relationship between HAW and work engagement is stronger (*B* = 1.34) than for those showing lower levels of interest-taking (*B* = 0.67). Thus, Hypothesis 3b is supported. Interest-taking moderates the positive relationship between HAW and newcomer work engagement.

Consequently, Hypothesis 4.1b and Hypothesis 4.2b predicted that interest-taking moderates the respective indirect effects of HAW on adaptive performance and OBC via work engagement. Multilevel estimates provided evidence for a moderation of the indirect effects. For values of the moderator at one standard deviation above and below the mean, results indicate conditional indirect effects of HAW on adaptive performance (*B* = 0.25 for interest-taking at −1SD, *B* = 0.50 for interest-taking at +1SD) and OCB (*B* = 0.08 for interest-taking at −1SD, *B* = 0.15 for interest-taking at +1SD) via work engagement (see Table 7). Thus, interest-taking moderates both indirect effects of HAW on adaptive performance (Hypothesis 4.1b) and OCB (Hypothesis 4.2b) via work engagement among newcomers.

#### 5.2.3 Supplementary analysis

As in Study 1, we conducted an additional analysis on the potential moderating effects of the other two subscales of the index of autonomous functioning. Again, no interaction of HAW with susceptibility to control was found [*B* = 0.10, 95&-CrI = [−0.21; 0.40]] among newcomers. Regarding the interaction of HAW and the subscale of authorship, multilevel estimates support an interaction effect on work engagement [*B* = 0.56, 95%-CrI = [0.24; 0.87]]. As the current study focuses on interest-taking, there will be no detailed discussion regarding self-congruence specifically. Nevertheless, potential implications for future research will be discussed later.

### 5.3 Discussion of study 2

In line with Study 1, all hypotheses regarding the mediating role of work engagement were supported among our newcomer sample. We found that HAW relates to work engagement, which further leads to increases in socialization outcomes. Therefore, our findings further support the proposition of HAW as an important resource for experienced employees and newcomers. In addition, and in contrast to Study 1, we were able to show that interest-taking moderates the positive effect of HAW-on-work engagement. This further highlights the importance of additionally considering interactions between job resources and personal resources and adds to our understanding of how individuals might profit from HAW. Also, the findings of our second study address the supposition of the newcomer pathway to organizational socialization (Saks and Gruman, 2018), which adapts the motivational process of the JD-R model and extends it to organizational socialization research. Finally, work engagement mediates the positive relationship of HAW with adaptive performance and OCB, respectively. Thus, by proving that work engagement is an important mediator between socialization resources (here: HAW) and socialization outcomes (here: adaptive performance and OCB), our findings add to the knowledge about the role of work engagement among newcomers during organizational socialization.

## 6 General discussion

To the best of our knowledge, this is the first study that investigates the relationship between HAW and adaptive performance or OCB among different populations of employees and, based on the JD-R model (Bakker and Demerouti, 2017), explains these relationships via work engagement. In particular, we examine the interactive effect of HAW and interest-taking on work engagement, ultimately predicting adaptative performance and OCB, through two studies with employees at different stages of their organizational careers: experienced employees (Study 1) and newcomers (Study 2). First, our results demonstrate that HAW is an important job resource for both experienced employees and newcomers, affecting work engagement and the motivational process of the JD-R model. Second, we confirmed the moderating role of interest-taking among newcomers, introducing interest-taking as a valuable personal resource that helps individuals benefit from HAW even more regarding their engagement. Third, both studies improve our understanding of the link between HAW and important work-related performance outcomes, namely adaptive performance and OCB. Based on JD-R theory, we demonstrate the crucial role of work engagement as a mediator with additional emphasis on organizational socialization research.

### 6.1 Theoretical contribution

We make several contributions to theory and research. First, we contribute to research on HAW regarding its role as a resource and its consequences for organizations and employees at different career stages. Recent research shows HAW is inherently connected with motivating job characteristics (Oerlemans and Bakker, 2018) and modern work environments (Kortsch et al., 2022). HAW is determined by the factors of self-actualization, meaningfulness, and community (Rehwaldt, 2017; Rehwaldt and Kortsch, 2022) and is supposed to relate to creativity, motivation, and performance (Rehwaldt, 2020). Implementing this conceptualization of HAW, we expand the knowledge about its relationship with motivation and performance among employees at different stages of their careers. Referring to the JD-R model's motivation process, we demonstrate that HAW is an important job resource (Bakker and Demerouti, 2007, 2017) for experienced employees and newcomers. By conducting two longitudinal studies among heterogeneous samples, we further address recent calls for longitudinal examinations of HAW and its consequences among diverse occupational groups (Rehwaldt and Kortsch, 2022).

By including interest-taking as a moderator in the relationship between HAW and work engagement, we further add to the understanding of how personal resources help individuals leverage their job resources more effectively. The JD-R model expects individuals with more personal resources to have better access to job resources, which will benefit the motivational process (Bakker et al., 2023). In our second study, we can show that newcomers with higher levels of interest-taking, compared to those with lower levels, are better able to benefit from the job resource HAW, such that they exhibit higher levels of work engagement. In doing so, we also respond to calls by Saks and Gruman (2012, 2018) to examine the joint effects of resources on work engagement among newcomers and contribute to organizational socialization literature. As mentioned earlier, we did not find a moderating effect of interest-taking among experienced employees. However, previous research has shown differences between newcomers and experienced employees in terms of the influence of personality on performance (e.g., Bauer et al., 2007; Tracey et al., 2007). Our results were surprising as we hypothesized that interest-taking benefits newcomers and experienced employees. One possible explanation could be the different phases and situations that lead to different perceptions of the work environment among new hires and experienced employees. While newcomers gain many new impressions, experienced employees have more expertise and experience to react to situational work events without much effort and attention. Therefore, because of their stage, newcomers benefit more from higher interest-taking, such as finding meaning in events and staying motivated. However, more research is needed to replicate and confirm these findings. The person x situation approach of JD-R theory by Bakker et al. (2023) can serve as a theoretical foundation to dig deeper into understanding these relationships.

We further contribute to the work engagement literature by clarifying the role of work engagement for newcomers during organizational socialization and experienced employees. In line with the JD-R model and numerous empirical research that suggests work engagement mediates the motivational process between job resources and performance outcomes (e.g., Bakker and Demerouti, 2017), our results show that work engagement fully mediates the relationship between HAW and adaptive performance and OCB, respectively. Regarding research on organizational socialization, our study adds to the limited knowledge about newcomer work engagement (Saks and Gruman, 2012, 2018). We introduce HAW as a valuable socialization resource and empirically support the mediating role of work engagement for newcomers.

Furthermore, our studies focus on adaptive performance and OCB and contribute to the knowledge of how to promote both simultaneously. Employees are confronted with changes in their work environment and show adaptive behavior to respond to those changes in their job tasks (Jundt et al., 2015). We contribute to the literature and expand the evidence on how engagement improves adaptive performance in employees, as there are only a few studies to link them (e.g., Kaya and Karatepe, 2020; Park et al., 2020). Furthermore, our results add to the existing literature on the relationship between work engagement and OCB (e.g., Borst et al., 2020; Gupta et al., 2017). Consistent with previous research, our results suggest a positive relationship between work engagement and OCB for both experienced employees and newcomers. Making an important contribution to organizational socialization research, this is the first study to demonstrate the relationship between newcomers' work engagement, adaptive performance, and OCB.

### 6.2 Practical implications

Current research suggests that HAW can provide valuable indicators for assessing progress and change in various work domains, such as employee acquisition, onboarding, and retention (e.g., Rehwaldt, 2017; Kortsch et al., 2022). Results from both studies extend and support these approaches and serve as a foundation for practical implementation for organizations seeking to build and improve HAW and work engagement from the outset and throughout employment. Creating favorable and inspiring work conditions and environments is strongly related to better performance (e.g., Bashir et al., 2020). Promoting HAW as a strategic HR measure has been shown to positively impact innovative performance and OCB (Fröhlich et al., 2025a,b).

Organizations should aim to build a professional and trusting community and empower employees to contribute to the bigger picture to strengthen HAW. This can be achieved, for example, by emphasizing the factors of meaningfulness and self-actualization, encouraging early employee participation, supporting open communication, implementing feedback, and promoting autonomous working (e.g., Rehwaldt, 2017; Kortsch et al., 2022). HR professionals and leaders can invest in targeted measures that strengthen meaning, self-actualization, and community in the workplace, e.g., via positive and supportive leadership (Fröhlich et al., 2025a,b), a culture of appreciation, or meaningful work tasks. In particular, training on positive leadership and coaching on HAW could help employees improve the factors of HAW. Companies should also provide employees with opportunities to improve their ability to align their actions with their interests to promote HAW and improve work engagement and extra-productive behaviors. Furthermore, training employees in interest-taking supports them in developing important skills and improving their experience of HAW. Implementing interventions addressing work engagement (see also Knight et al., 2019) and HAW is a promising avenue for future research.

### 6.3 Limitations and avenues for future research

First, we used self-report data, susceptible to certain biases (e.g., social desirability) and inflated associations due to common method bias. We addressed this issue by using validated and distinct measures for all focal constructs and carefully designing the studies (e.g., separating measurements during the day in Study 1). Furthermore, multilevel CFAs confirmed the distinctiveness of the focal constructs, and a supplemental Harman's single-factor test suggests the absence of common method variance. However, to further mitigate potential biases, future research could include, for example, other sources of external information or ratings from team members or supervisors. Furthermore, given the nature of our approach, we focused on the investigation of the effects in the direction of HAW on work engagement in a multilevel design. Future studies could apply multilevel modeling to integrate further constructs that might explain our proposed relationships (for further factors, see the second limitation as well), even at the team- or organizational level, or implement cross-lagged panel models to investigate potential feedback-loops that might lead from engagement to HAW.

Second, especially in the first study, there is no significant moderator effect, raising two questions: (1) Which moderators could further explain the link between HAW and work engagement, especially for experienced employees? We suggest that the lack of effectiveness of interest-taking as a moderator among experienced employees might be due to them already being accustomed to their work environment. However, future studies might test this supposition. Also, exploring alternative personal resources as moderators (e.g., workplace curiosity or proactive personality) or additional factors such as job autonomy or role clarity, might be a fruitful avenue for future studies. Weinstein et al. (2012) found associations of interest-taking with the Big Five personality traits. Thus, further investigation into how personality traits impact the effectiveness of interest-taking or affect the link between HAW and engagement could benefit our understanding of the relationship. These studies might shed light on the question of how more self-regulated personality profiles could generally benefit more from HAW than others. (2) Are there possibly industry or organizational differences that explain how interest-taking or personality traits in some industries interacts more with HAW and thus sets motivational processes in motion that favor individual work engagement? For example, organizational culture, workplace policies, or job complexity could form contextual factors that help further explain the relationships. Similarly, although we acquired two heterogeneous samples that each integrated multiple industries and jobs, our results are based on so-called WEIRD (i.e., from Western, Educated, Industrialized, Rich, and Democratic societies) samples. Investigating the HAW-engagement-performance link in various working areas, industries, and cultures would be a promising avenue for future research.

Third, it should also be noted that a large portion of the first study was collected during the Corona pandemic, which is an additional limitation. In addition, future intervention studies could examine how training or coaching, focusing on work engagement or factors for HAW, enhances extra-productive behavior.

## Data Availability

The datasets presented in this study can be found in online repositories. The names of the repository/repositories and accession number(s) can be found below: OSF repository at https://osf.io/ed9g7/?view_only=e6633b8e191c4d2fb4941d8d2a5d5e4b.

## Footnotes

^1^For more details on the framework of hedonic and eudaimonic well-being we refer interested readers to: Ryan and Deci (2001) and Delle Fave (2023).

^2^Despite significant correlations of age and work experience with OCB and of organizational tenure with interest-taking, we excluded these control variables from further analyses in order to minimize power reduction associated with type II error inflation (Becker, 2005; Becker et al., 2016). Supplemental analyses that accounted for the aforementioned variables also did not reveal a different pattern of results.

^3^As in Study 1 and due to nonsignificant correlations with the focal variables, additional control variables are excluded further analyses.

## References

[B1] Allan B. A. Batz-Barbarich C. Sterling H. M. Tay L. (2019). Outcomes of meaningful work: a meta-analysis. J. Manag. Stud. 56, 500–528. 10.1111/joms.12406

[B2] Asparouhov T. Muthén B. (2021a). Advances in bayesian model fit evaluation for structural equation models. Struct. Equat. Model. Multidiscipl. J. 28, 1–14. 10.1080/10705511.2020.1764360

[B3] Asparouhov T. Muthén B. (2021b). Bayesian estimation of single and multilevel models with latent variable interactions. Struct. Equat. Model. Multidiscipl. J. 28, 314–328. 10.1080/10705511.2020.1761808

[B4] Bakker A. B. Albrecht S. L. Leiter M. P. (2011). Key questions regarding work engagement. Eur. J. Work Organiz. Psychol. 20, 4–28. 10.1080/1359432X.2010.485352

[B5] Bakker A. B. Demerouti E. (2007). The job demands-resources model: state of the art. J. Manag. Psych 22, 309–328. 10.1108/0268394071073311531861812

[B6] Bakker A. B. Demerouti E. (2017). Job demands-resources theory: taking stock and looking forward. J. Occup. Health Psychol. 22, 273–285. 10.1037/ocp0000056 27732008

[B7] Bakker A. B. Demerouti E. (2024). Job demands-resources theory: frequently asked questions. J. Occup. Health Psychol. 29, 188–200. 10.1037/ocp0000376 38913705

[B8] Bakker A. B. Demerouti E. Sanz-Vergel A. (2023). Job demands–resources theory: ten years later. Annu. Rev. Organ. Psychol. Organ. Behav. 10, 25–53. 10.1146/annurev-orgpsych-120920-053933

[B9] Bakker A. B. Oerlemans W. G. M. (2016). Momentary work happiness as a function of enduring burnout and work engagement. J. Psychol. 150, 755–778. 10.1080/00223980.2016.1182888 27223847

[B10] Baruch-Feldman C. Brondolo E. Ben-Dayan D. Schwartz J. (2002). Sources of social support and burnout, job satisfaction, and productivity. J. Occup. Health Psychol. 7, 84–93. 10.1037/1076-8998.7.1.84 11827236

[B11] Bashir A. Amir A. Jawaad M. Hasan T. (2020). Work conditions and job performance: an indirect conditional effect of motivation. Cogent Business Manag. 7:1801961. 10.1080/23311975.2020.1801961

[B12] Bauer T. N. Bodner T. Erdogan B. Truxillo D. M. Tucker J. S. (2007). Newcomer adjustment during organizational socialization: a meta-analytic review of antecedents, outcomes, and methods. J. Appl. Psychol. 92, 707–721. 10.1037/0021-9010.92.3.707 17484552

[B13] Bauer T. N. Erdogan B. (2011). “Organizational socialization: the effective onboarding of new employees,” in APA Handbook of Industrial and Organizational Psychology, Vol 3: Maintaining, Expanding, and Contracting the Organization, ed. Zedeck S. (Washington, DC: American Psychological Association), 51–64. 10.1037/12171-002

[B14] Becker T. E. (2005). Potential problems in the statistical control of variables in organizational research: a qualitative analysis with recommendations. Organ. Res. Methods 8, 274–289. 10.1177/109442810527802127409075

[B15] Becker T. E. Atinc G. Breaugh J. A. Carlson K. D. Edwards J. R. Spector P. E. (2016). Statistical control in correlational studies: 10 essential recommendations for organizational researchers. J. Organ. Behav. 37, 157–167. 10.1002/job.2053

[B16] Borst R. T. Kruyen P. M. Lako C. J. Vries M. S. (2020). The attitudinal, behavioral, and performance outcomes of work engagement: a comparative meta-analysis across the public, semipublic, and private sector. Rev. Public Pers. Administr. 40, 613–640. 10.1177/0734371X19840399

[B17] Breevaart K. Bakker A. B. Demerouti E. Sleebos D. M. Maduro V. (2014). Uncovering the underlying relationship between transformational leaders and followers' task performance. J. Pers. Psychol. 13, 194–203. 10.1027/1866-5888/a000118

[B18] Butler J. Kern M. L. (2016). The PERMA-Profiler: a brief multidimensional measure of flourishing. Intnl. J. Wellbeing 6, 1–48. 10.5502/ijw.v6i3.52638015927

[B19] Cheese P. (2008). Driving high performance in the talent-powered organization. Strat. HR Rev. 7, 25–31. 10.1108/14754390810880507

[B20] Chiaburu D. S. Oh I.-S. Stoverink A. C. Park H. Bradley C. Barros-Rivera B. A. (2022). Happy to help, happy to change? A meta-analysis of major predictors of affiliative and change-oriented organizational citizenship behaviors. J. Vocat. Behav. 132;103664. 10.1016/j.jvb.2021.103664

[B21] Christian M. S. Garza A. S. Slaughter J. E. (2011). Work engagement: a quantitative review and test of its relations with task and contextual performance. Pers. Psychol. 64, 89–136. 10.1111/j.1744-6570.2010.01203.x

[B22] Costa P. L. Passos A. M. Bakker A. B. (2016). The work engagement grid: predicting engagement from two core dimensions. J. Manag. Psych 31, 774–789. 10.1108/JMP-11-2014-0336

[B23] Delle Fave A. (2023). “Eudaimonic and hedonic happiness,” in Encyclopedia of Quality of Life and Well-Being Research, 2nd Edn., ed. Maggino F. (Cham: Springer International Publishing; Imprint Springer), 2206–2212. 10.1007/978-3-031-17299-1_3778

[B24] Ehresmann C. Badura B. (2018). “Sinnquellen in der Arbeitswelt und ihre Bedeutung für die Gesundheit [Sources of meaning in the world of work and their significance for health],” in Fehlzeiten-Report 2018: Sinn erleben - Arbeit und Gesundheit; Zahlen, Daten, Analysen aus allen Branchen der Wirtschaft; 250 Tabellen, eds. Badura B. Ducki A. Schröder H. Klose J. Meyer M. (Berlin, Germany: Springer), 47–59. 10.1007/978-3-662-57388-4_4

[B25] Eldor L. Harpaz I. (2016). A process model of employee engagement: the learning climate and its relationship with extra-role performance behaviors. J. Organ. Behav. 37, 213–235. 10.1002/job.2037

[B26] Enders C. K. Tofighi D. (2007). Centering predictor variables in cross-sectional multilevel models: a new look at an old issue. Psychol. Methods 12, 121–138. 10.1037/1082-989X.12.2.121 17563168

[B27] Fang J. Wen Z. Hau K.-T. (2019). Mediation effects in 2-1-1 multilevel model: evaluation of alternative estimation methods. Struct. Equat. Model. Multidiscipl. J. 26, 591–606. 10.1080/10705511.2018.1547967

[B28] Fox S. Spector P. E. Goh A. Bruursema K. Kessler S. R. (2012). The deviant citizen: measuring potential positive relations between counterproductive work behaviour and organizational citizenship behaviour. J. Occup. Organ. Psychol. 85, 199–220. 10.1111/j.2044-8325.2011.02032.x

[B29] Fröhlich P. Rehwaldt R. Beitz S. Diestel S. Kortsch T. (2025a). Leadership, happiness, and extra-role behavior: the role of happiness at work as a mediator between instrumental leadership and OCB in newcomers. Gr Interakt Org. 10.1007/s11612-025-00809-0. [Epub ahead of print].

[B30] Fröhlich P. Rehwaldt R. Kortsch T. Radaca E. Diestel S. (2025b). Newcomers' happiness at work trajectories and their relation to servant leadership and innovative performance. Eur. J. Work Organiz. Psychol. 10.1080/1359432X.2025.2495988. [Epub ahead of print].

[B31] Green P. I. Finkel E. J. Fitzsimons G. M. Gino F. (2017). The energizing nature of work engagement: toward a new need-based theory of work motivation. Res. Organiz. Behav. 37, 1–18. 10.1016/j.riob.2017.10.007

[B32] Griffin M. A. Neal A. Parker S. K. (2007). A new model of work role performance: positive behavior in uncertain and interdependent contexts. AMJ 50, 327–347. 10.5465/amj.2007.2463443833343446

[B33] Gupta M. Shaheen M. Reddy P. K. (2017). Impact of psychological capital on organizational citizenship behavior. JMD 36, 973–983. 10.1108/JMD-06-2016-0084

[B34] Hafeez S. Memon M. A. Mirza M. Z. Raziq M. M. Sarwar N. Ting H. (2024). The dual impact of job variety on employee happiness and stress: the mediating role of employee engagement and burnout. JMD 43, 170–186. 10.1108/JMD-03-2023-0084

[B35] Halbesleben J. R. B. Neveu J.-P. Paustian-Underdahl S. C. Westman M. (2014). Getting to the “COR”. J. Manag. 40, 1334–1364. 10.1177/0149206314527130

[B36] Hobfoll S. E. Johnson R. J. Ennis N. Jackson A. P. (2003). Resource loss, resource gain, and emotional outcomes among inner city women. J. Pers. Soc. Psychol. 84, 632–643. 10.1037/0022-3514.84.3.632 12635922

[B37] Jundt D. K. Shoss M. K. Huang J. L. (2015). Individual adaptive performance in organizations: a review. J. Organ. Behav. 36, S53–S71. 10.1002/job.1955

[B38] Kanjanakan P. Zhu D. Doan T. Kim P. B. (2021). Taking stock: a meta-analysis of work engagement in the hospitality and tourism context. J. Hosp. Tourism Res. 47, 851–876. 10.1177/10963480211066958

[B39] Kaya B. Karatepe O. M. (2020). Does servant leadership better explain work engagement, career satisfaction and adaptive performance than authentic leadership? IJCHM 32, 2075–2095. 10.1108/IJCHM-05-2019-0438

[B40] Knight C. Patterson M. Dawson J. (2019). Work engagement interventions can be effective: a systematic review. Eur. J. Work Organiz. Psychol. 28, 348–372. 10.1080/1359432X.2019.1588887

[B41] Kortsch T. Rehwaldt R. Schwake M. E. Licari C. (2022). Does remote work make people happy? Effects of flexibilization of work location and working hours on happiness at work and affective commitment in the german banking sector. IJERPH 19:9117. 10.3390/ijerph19159117 35897480 PMC9368397

[B42] Lesener T. Gusy B. Jochmann A. Wolter C. (2020). The drivers of work engagement: a meta-analytic review of longitudinal evidence. Work Stress 34, 259–278. 10.1080/02678373.2019.1686440

[B43] Lesener T. Gusy B. Wolter C. (2019). The job demands-resources model: a meta-analytic review of longitudinal studies. Work Stress 33, 76–103. 10.1080/02678373.2018.1529065

[B44] Marsh H. W. Dicke T. Riley P. Parker P. D. Guo J. Basarkod G.. (2023). School principals' mental health and well-being under threat: a longitudinal analysis of workplace demands, resources, burnout, and well-being. Appl. Psychol. Health Wellbeing 15, 999–1027. 10.1111/aphw.12423 36504371

[B45] Mathumbu D. Dodd N. (2013). Perceived organisational support, work engagement and organisational citizenship behaviour of nurses at victoria hospital. J. Psychol. 4, 87–93. 10.1080/09764224.2013.11885497

[B46] Meijman T. F. Mulder G. (1998). “Psychological aspects of workload,” in Handbook of Work and Organizational Psychology: Work psychology, eds. Drenth P. J. D. Thierry H. (Hove: Palgrave Macmillan, London), 5–33.

[B47] Monteiro B. Santos V. Reis I. Sampaio M. C. Sousa B. Martinho F.. (2020). Employer branding applied to SMEs: a pioneering model proposal for attracting and retaining talent. Information 11:574. 10.3390/info11120574

[B48] Muthén L. K. Muthén B. (2017). Mplus User's Guide, 8th Ed.

[B49] Neuber L. Englitz C. Schulte N. Forthmann B. Holling H. (2022). How work engagement relates to performance and absenteeism: a meta-analysis. Eur. J. Work Organiz. Psychol. 31, 292–315. 10.1080/1359432X.2021.195398937239689

[B50] Neuberger O. (2006). Mikropolitik und Moral in Organisationen: Herausforderung der Ordnung [Micropolitics and Morality in Organizations: The Challenge of Order], 2nd Edn. München; Stuttgart: Lucius and Lucius.

[B51] Oerlemans W. G. M. Bakker A. B. (2018). Motivating job characteristics and happiness at work: a multilevel perspective. J. Appl. Psychol. 103, 1230–1241. 10.1037/apl0000318 29963893

[B52] Organ D. W. (1997). Organizational citizenship behavior: it's construct clean-up time. Hum. Perform. 10, 85–97. 10.1207/s15327043hup1002_2

[B53] Park Y. Lim D. H. Kim W. Kang H. (2020). Organizational support and adaptive performance: the revolving structural relationships between job crafting, work engagement, and adaptive performance. Sustainability 12:4872. 10.3390/su12124872

[B54] Podsakoff P. M. MacKenzie S. B. Lee J.-Y. Podsakoff N. P. (2003). Common method biases in behavioral research: a critical review of the literature and recommended remedies. J. Appl. Psychol. 88, 879–903. 10.1037/0021-9010.88.5.879 14516251

[B55] Podsakoff P. M. MacKenzie S. B. Paine J. B. Bachrach D. G. (2000). Organizational citizenship behaviors: a critical review of the theoretical and empirical literature and suggestions for future research. J. Manag. 26, 513–563. 10.1177/014920630002600307

[B56] Preacher K. J. Curran P. J. Bauer D. J. (2006). Computational tools for probing interactions in multiple linear regression, multilevel modeling, and latent curve analysis. J. Educ. Behav. Stat. 31, 437–448. 10.3102/1076998603100443738293548

[B57] Preacher K. J. Zhang Z. Zyphur M. J. (2011). Alternative methods for assessing mediation in multilevel data: the advantages of multilevel SEM. Struct. Equat. Model. Multidiscipl. J. 18, 161–182. 10.1080/10705511.2011.55732926741527

[B58] Preacher K. J. Zyphur M. J. Zhang Z. (2010). A general multilevel SEM framework for assessing multilevel mediation. Psychol. Methods 15, 209–233. 10.1037/a0020141 20822249

[B59] Pryce-Jones J. Lindsay J. (2014). What happiness at work is and how to use it. ICT 46, 130–134. 10.1108/ICT-10-2013-0072

[B60] Radaca E. Diestel S. (2025). The role of trait self-regulation and innovative team climate in reducing daily workplace conflicts initiated via emotional dissonance. Gr Interakt Org. 10.1007/s11612-025-00817-0. [Epub ahead of print].

[B61] Rehwaldt R. (2017). Die glückliche Organisation [The Happy Organization]. Wiesbaden: Springer Fachmedien Wiesbaden. 10.1007/978-3-658-19251-817511396

[B62] Rehwaldt R. (2020). Das arbeitsglück messen [measureing happiness of work]. Control Manag. Rev. 64, 64–69. 10.1007/s12176-020-0097-3

[B63] Rehwaldt R. Kortsch T. (2022). Was macht bei der Arbeit glücklich? Entwicklung und Validierung einer mehrdimensionalen Skala zur Erfassung von Glück bei der Arbeit [What makes you happy at work? Development and validation of a multidimensional scale to capture happiness at work]. Zeitschrift Arbeits- Organisationspsychol. A&O 66, 72–86. 10.1026/0932-4089/a000373

[B64] Rodríguez-Muñoz A. Sanz-Vergel A. I. (2013). Happiness and well-being at work: a special issue introduction. Rev. Psicol. Trabajo Organiz. 29, 95–97. 10.5093/tr2013a14

[B65] Rossberg J. I. Eiring Ø. Friss S. (2004). Work environment and job satisfaction. Soc. Psychiatry Psychiatr. Epidemiol. 39, 576–580. 10.1007/s00127-004-0791-z 15243696

[B66] Ryan R. M. Deci E. L. (2001). On happiness and human potentials: a review of research on hedonic and eudaimonic well-being. Annu. Rev. Psychol. 52, 141–166. 10.1146/annurev.psych.52.1.141 11148302

[B67] Ryan R. M. Deci E. L. (2008). A self-determination theory approach to psychotherapy: the motivational basis for effective change. Can. Psychol. 49, 186–193. 10.1037/a0012753

[B68] Saks A. M. (2019). Antecedents and consequences of employee engagement revisited. J. Org. Effect. 6, 19–38. 10.1108/JOEPP-06-2018-0034

[B69] Saks A. M. Gruman J. A. (2012). “Getting newcomers on board: a review of socialization practices and introduction to socialization resources theory,” in The Oxford Handbook of Organizational Socialization, ed. Wanberg C. R. (Oxford: Oxford Univ. Press), 27–55. 10.1093/oxfordhb/9780199763672.013.0003

[B70] Saks A. M. Gruman J. A. (2018). Socialization resources theory and newcomers' work engagement. A new pathway to newcomer socialization. Career Dev. Int. 23, 12–32. 10.1108/CDI-12-2016-0214

[B71] Salas-Vallina A. Alegre J. Fernandez R. (2017). Happiness at work and organisational citizenship behaviour. IJM 38, 470–488. 10.1108/IJM-10-2015-0163 27329503

[B72] Salas-Vallina A. Pozo-Hidalgo M. Gil-Monte P. R. (2020). Are happy workers more productive? The mediating role of service-skill use. Front. Psychol. 11:456. 10.3389/fpsyg.2020.00456 32292366 PMC7120033

[B73] Schaufeli W. B. Bakker A. B. Salanova M. (2006). The measurement of work engagement with a short questionnaire: a cross-national study. Educ. Psychol. Meas. 66, 701–716. 10.1177/0013164405282471

[B74] Schaufeli W. B. Salanova M. González-roma V. Bakker A. B. (2002). The measurement of engagement and burnout: a two sample confirmatory factor analytic approach. J. Happiness Stud. 3, 71–92. 10.1023/A:1015630930326

[B75] Singh A. Banerji R. (2022). Happiness at work, organization citizenship behaviour and workplace diversity: a study on Indian private sector bank employees. ICT 54, 460–475. 10.1108/ICT-05-2021-0037

[B76] Spector P. E. Bauer J. A. Fox S. (2010). Measurement artifacts in the assessment of counterproductive work behavior and organizational citizenship behavior: do we know what we think we know? J. Appl. Psychol. 95, 781–790. 10.1037/a0019477 20604597

[B77] Staufenbiel T. Hartz C. (2000). Organizational citizenship behavior: entwicklung und erste validierung eines messinstruments. Diagnostica 46, 73–83. 10.1026//0012-1924.46.2.73

[B78] Su R. Tay L. Diener E. (2014). The development and validation of the Comprehensive Inventory of Thriving (CIT) and the Brief Inventory of Thriving (BIT). Appl. Psychol. Health Wellbeing 6, 251–279. 10.1111/aphw.12027 24919454

[B79] Sulea C. Virga D. Maricutoiu L. P. Schaufeli W. Zaborila Dumitru C. Sava F. A. (2012). Work engagement as mediator between job characteristics and positive and negative extra-role behaviors. Career Dev Int 17, 188–207. 10.1108/13620431211241054

[B80] Thompson A. Bruk-Lee V. (2021). Employee happiness: why we should care. Appl. Res. Qual. Life 16, 1419–1437. 10.1007/s11482-019-09807-z

[B81] Tracey J. B. Sturman M. C. Tews M. J. (2007). Ability versus personality. Cornell Hotel Restaur. Administr. Quart. 48, 313–322. 10.1177/0010880407302048

[B82] van Maanen J. E. Schein E. H. (1979). Toward a theory of organizational socialization. Res. Organiz. Behav. 1, 209–264.

[B83] Wang L. Preacher K. J. (2015). Moderated mediation analysis using bayesian methods. Struct. Equat. Model. Multidiscipl. J. 22, 249–263. 10.1080/10705511.2014.935256

[B84] Weinstein N. Przybylski A. K. Ryan R. M. (2012). The index of autonomous functioning: development of a scale of human autonomy. J. Res. Pers. 46, 397–413. 10.1016/j.jrp.2012.03.007

[B85] Zyphur M. J. Oswald F. L. (2015). Bayesian estimation and inference. A user's guide. J. Manag. 41, 390–420. 10.1177/0149206313501200

